# A spatio temporal spectral framework for plant stress phenotyping

**DOI:** 10.1186/s13007-019-0398-8

**Published:** 2019-02-06

**Authors:** Raghav Khanna, Lukas Schmid, Achim Walter, Juan Nieto, Roland Siegwart, Frank Liebisch

**Affiliations:** 1 Autonomous Systems Lab, ETH Zürich, Leonhardstrasse 21, Zurich, Switzerland; 2 Crop Science Group, Department of Environmental Systems Science, ETH Zürich, Zurich, Switzerland

**Keywords:** Phenotyping, Plant-stress, Nitrogen, Weed, Water, Dataset, Multispectral, 3D

## Abstract

**Background:**

Recent advances in high throughput phenotyping have made it possible to collect large datasets following plant growth and development over time, and those in machine learning have made inferring phenotypic plant traits from such datasets possible. However, there remains a dirth of datasets following plant growth under stress conditions along with methods for inferring them using only remotely sensed data, especially under a combination of multiple stress factors such as drought, weeds and nutrient deficiency. Such stress factors and their combinations are commonly encountered during crop production and being able to accurately detect and treat such stress conditions in an automated and timely manner can provide a major boost to farm yields with minimal resource input.

**Results:**

We present a generic framework for remote plant stress phenotyping that consists of a dataset with spatio-temporal-spectral data following sugarbeet crop growth under optimal, drought, low and surplus nitrogen fertilization, and weed stress conditions, along with a machine learning based methodology for systematically inferring these stress conditions from the remotely measured data. The dataset contains biweekly color images, infra-red stereo image pairs and hyperspectral camera images along with applied treatment parameters and environmental factors like temperature and humidity, collected over two months. We present a plant agnostic methodology for deriving plant trait indicators such as canopy cover, height, hyperspectral reflectance and vegetation indices along with a spectral 3D reconstruction of the plants from the raw data to serve as a benchmark. Additionally, we provide fresh and dry weight measurements for both the above (canopy) and below (beet) ground biomass at the end of the growing period to serve as indicators of expected yield. We further describe a data driven, machine learning based method to infer water, Nitrogen and weed stress using the derived plant trait indicators. We use the plant trait indicators to evaluate 8 different classification approaches from which the best classifier achieved a mean cross validation accuracy of $\approx$ 93, 76 and 83% for drought, nitrogen and weed stress severity classification respectively. We also show that our multi-modal approach significantly improves classifier performance over using any single modality.

**Conclusion:**

The presented framework and dataset can serve as a valuable reference for creating and comparing processing pipelines which extract plant trait indicators and infer prevalent stress factors from remote sensing data under a variety of environments and cropping conditions. These techniques can then be deployed on farm machinery or robots enabling automated, precise and timely corrective interventions for maximising yield.

## Introduction

Plants grown in most crop production fields and breeding nurseries suffer from varying types and severities of biotic and abiotic stresses such as nutrient deficiency and weed pressure which have adverse affects on yields [3]. For precise and timely corrective intervention, a significant challenge is to determine the types as well as the severity levels of the multiple stress conditions present at different locations on the field. This information is important for field experiments as well as crop management in farmers’ fields. Once the type and severity level of each of the many possible stress factors can be accurately determined, corrective treatments such as irrigation, fertilizer and herbicide application can then be applied in a precise local manner, targeting only areas where these treatments would have a beneficial impact while simultaneously adjusting the applied amount to meet the actual demand. This concept is widely described as variable rate application (VRA) and is linked to increased resource use efficiency and economic benefit [4, 5]. Furthermore, the recent surge in interest in agricultural robotics [6–8], specifically pertaining to precision agriculture applications [9] make *automated, remote sensing based* plant stress inference, as a key capability of such systems, a pertinent challenge.

Today, determination of crop stress factors using visible symptoms is still often a manual and complex task predominantly carried out by trained and experienced individuals, such as agronomists, crop scientists and plant pathologists, since a variety of stress factors can manifest themselves through similar symptoms. Similar to plant breeding, however, the manual process is laborious, time-consuming and not always reproducible due to the inherently subjective nature of manual ratings, experience and interpretation [10]. Advancements in high throughput phenotyping, remote sensing hardware and machine learning software have now made remote sensing based plant stress inference computationally tractable [11]. State of the art machine learning methods [12, 13] can employ large amounts of multi-modal data to produce accurate classification and regression models that can be used for such inference tasks. There exist a variety of readily available, rich, open-source software libraries [14–16] with which, given suitable data, one can quickly iterate and determine the most suitable machine learning algorithm and create an accurate model for a given inference task. With the proliferation of these automated high throughput plant phenotyping tools, a large number of recent studies have been focused on studying plant growth in relation to genotype variety [17, 18] and under stress using a variety of sensors and their combinations [11]. Studies on plant stress include those on drought stress [19, 20], heat stress [21], salt stress [22, 23], nutrient deficiency [24, 25] and biotic stress [10, 12, 13, 26–28]. Sensor modalities used in these studies include colour, hyperspectral, thermal and fluorescence imaging. However, most studies typically include primarily one sensor modality [11, 29] and focuses on one out of the many aforementioned stress conditions [11, 30], which often occur simultaneously on real fields. These stress studies also typically only look at individual time points during plant development [11]. Studies covering the temporal range of plant growth may allow for better characterization of dynamic plant response to stress. There also remains a dirth of open, high quality, multi-modal datasets which can be used along with the powerful machine learning software libraries to develop, compare and benchmark methods for automated plant stress inference.

**Fig. 1:**
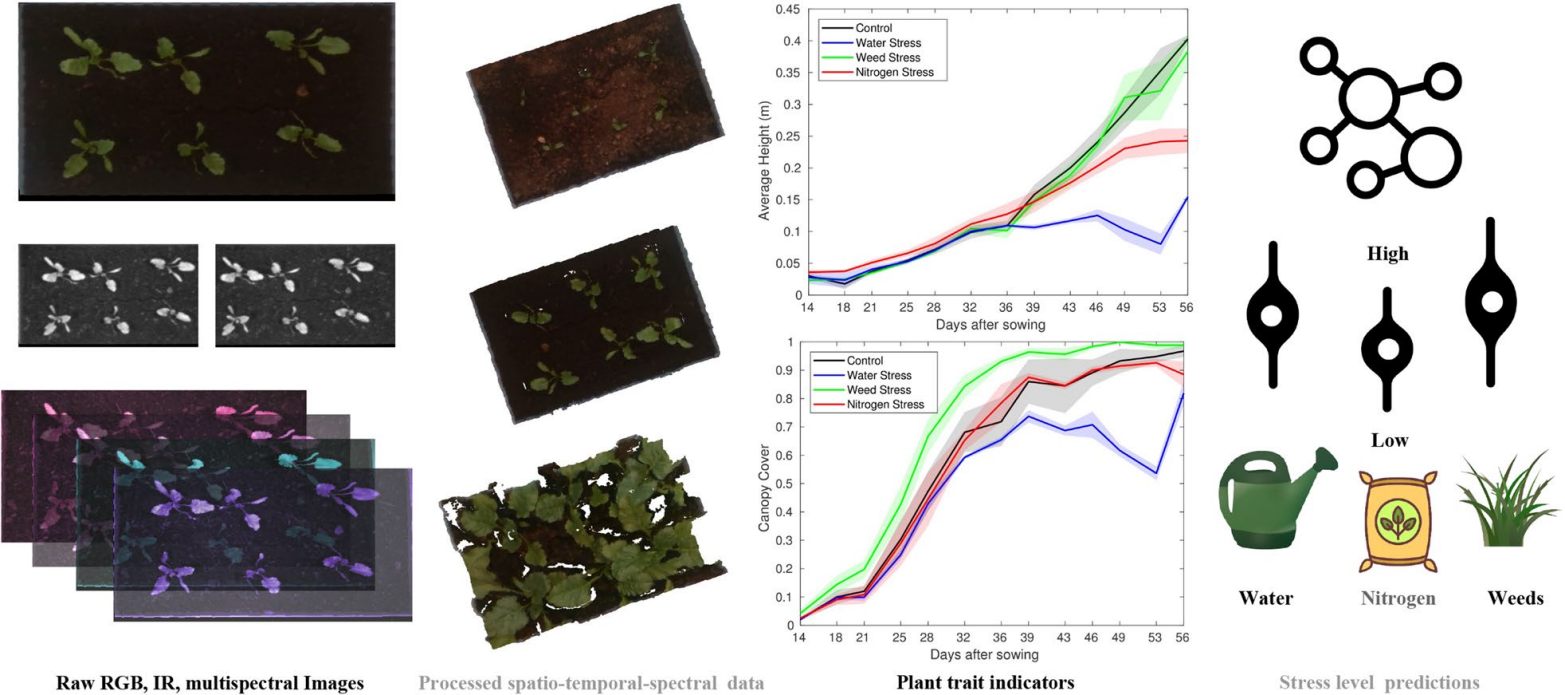
An overview of the framework described in this paper. This work presents a methodology for building and benchmarking machine learning models that can infer plant stress using remotely sensed, multi-modal data. Our framework consists of a spatio-temporal spectral dataset, image pre-processing and classification algorithms along with reference plant trait measurements and stress type and severity level labels to serve as ground truth. Our generic, plant agnostic pipeline starts with raw input imagery from RGB, stereo infrared (IR) and multispectral cameras, followed by pre-processing steps of vegetation segmentation, 3D reconstruction and reflectance normalization to transform this raw data into plant trait indicators such as canopy cover, average height and normalized narrow-band reflectances over time. We then train machine learning models which can use these indicators to predict severity levels for Water, Nitrogen and Weed stress simultaneously. We show the effectiveness of our framework by using the trained models to accurately predict stress severity levels on novel test data. We release the collected dataset [1] and accompanying pre-processing and classification software [2] under an open source license for the broader plant research community

To address these issues, we studied plant growth under different severities of 2 commonly occurring abiotic stress conditions-drought and nitrogen availability and one biotic stress-weeds. We imaged the plants subjected to multiple combinations of these stress conditions with color, stereo infrared and narrow-band hyperspectral cameras providing bi-weekly multi-modal information on the growth of sugarbeet plants under these stress conditions. We show how such a dataset can serve as an test bench for rapidly developing and evaluating classification models which determine the presence and severity of different stress factors using remotely sensed data. The primary contributions of this work are (Fig. 1):

- A open, publicly available dataset [1] from a two month long experiment consisting of biweekly RGB, stereo and hyperspectral imagery for sugar beet plants grown in a greenhouse subject to known severity levels of water, nitrogen and weed stress.
- Reference measurements including environmental temperature and humidity logs, along with applied treatment regimens creating the different stress conditions, SPAD measurements and harvested beet biomass after the experiment enabling the systematic study of the effects of different stress factors on plant development and yield.
- Generic, plant agnostic pre and post processing software [2] for the raw imagery, providing functionality for spectral point cloud generation and extraction of a variety of remote phenotypic plant trait indicators such as canopy cover, height and spectral vegetation indices along with an analysis of the impact of the different stress factors on the extracted plant trait indicators and biomass production.
- A machine learning based methodology, using the extracted plant trait indicators for simultaneous stress severity level classification of drought, nitrogen and weed stress, released as part of the open source software suite [2].

With this methodology, we show that with spatio-temporal spectral data it becomes possible to create accurate classification models for a variety of useful tasks, specifically, the simultaneous detection of the presence and severity of drought, nitrogen and weed stress. The same methodology can also be used to collect similar datasets for other plant species, which can then be used with the provided software suite to conveniently build similar models for a variety of different crops. These validated models can then be augmented with data from limited, less expensive field trials and deployed on hand-held sensor setups [31], Unmanned Aerial Vehicles (UAVs), smart tractors and Unmanned Ground Vehicles (UGVs) for use in plant breeding and sustainable crop production [6, 7].

## Materials and methods

### Plant cultivation and stress treatments

The experiment was conducted at the ETH research station for plant sciences in Lindau Eschikon, Switzerland. The sugar beet plants (*Beta vulgaris*) of the variety “Samuela” (KWS Suisse SA, Basel, Switzerland) were grown in a greenhouse chamber under controlled climate conditions—24/12 $({}^{\circ }\hbox {C})$ Day/Night temperature, a relative humidity of 50–80 percent (average 60%), and additional light using Eye Clean Arc MT 400DL/BH lamps with a color temperature of 6400 K when ambient radiation was below 25 klux. The achieved radiation intensity ranged approximately between 300 and 680 $\upmu \hbox {mol}/\hbox {cm}^{2}\hbox {s}$. The radiation angle between the cultivation light source and the plants was nadir $\pm 20^{\circ }$. Above 45 klux the shading screen was closed. For the experiment, 6 sugar beet plants each were sown on 18.01.2018 in 30 cultivation boxes of size 40 × 20 × 15.5 cm using a peat substrate (Klasmann substrate 1 and 2, Klasmann–Deilmann GmbH, Geeste, Germany). The plant boxes were placed on tables about 2 m from the artificial light sources. Regular watering was volume controlled and applied manually according to necessity two to three times a week. The experiment was conducted until 29.03.2018 when the plants were manually harvested.

In addition to the control group of plant cultivation boxes which were provided with sufficient nutrients and were not subjected to any weed pressure, we established a mix of three different stresses relevant for field crops in general and for sugar beet production in particular—Nitrogen (N) availability, weed pressure and water supply listed in Table 1. Different severity levels for each of these three stress types and their combinations were established which span the range of expected conditions which may be observed on the field.

**Table 1:** Overview of the experimental treatments

Treatment	# of Boxes	Soil type	Water input	Nitrogen input	Weed pressure
Low N	3	1	Sufficient	Low	None
Med N	3	2	Sufficient	Medium	None
High N	3	2	Sufficient	High	None
Med weeds	3	2	Sufficient	High	Medium
High weeds	3	2	Sufficient	High	High
Dry	3	2	Limited	Medium	None
Weed only dicot	1	2	Sufficient	High	High
Weed only monocot	1	2	Sufficient	High	High
Weed only mixed	1	2	Sufficient	High	High
Low N-med weed	3	1	Sufficient	Low	Medium
Drying-med N-high weed	3	2	Limited	Medium	High
Drying-low N	3	1	Limited	Low	None

30 boxes were monitored during the experiment consisting of 3 repetitions of a variety of treatments representing a range of stress factors and their severity levels commonly observed on the field

#### Nitrogen stress

For plant N availability, three levels: low, medium and high were established, targeting a deficient, sufficient and surplus N supply, simulating an N availability equivalent of 20, 40 and 80 kg/ha on the field respectively. These N supply values were chosen based on literature [6, 32, 33], own experience and local farmers’ best management practices. The low N supply was achieved using the Klasmann substrate 1 which contains minor amounts of N, sufficient only for initial plant growth. The medium N level was achieved using the Klasman substrate two containing amounts of N sufficient for 1–2 months of plant growth and the high level received additional N by means of fertigation once a week from 14.02.2018 onwards using 0.2 percent Wuxal Profi (Syngenta Agro AG, Dielsdorf, Switzerland) [34].

#### Weed stress

**Fig. 2:**
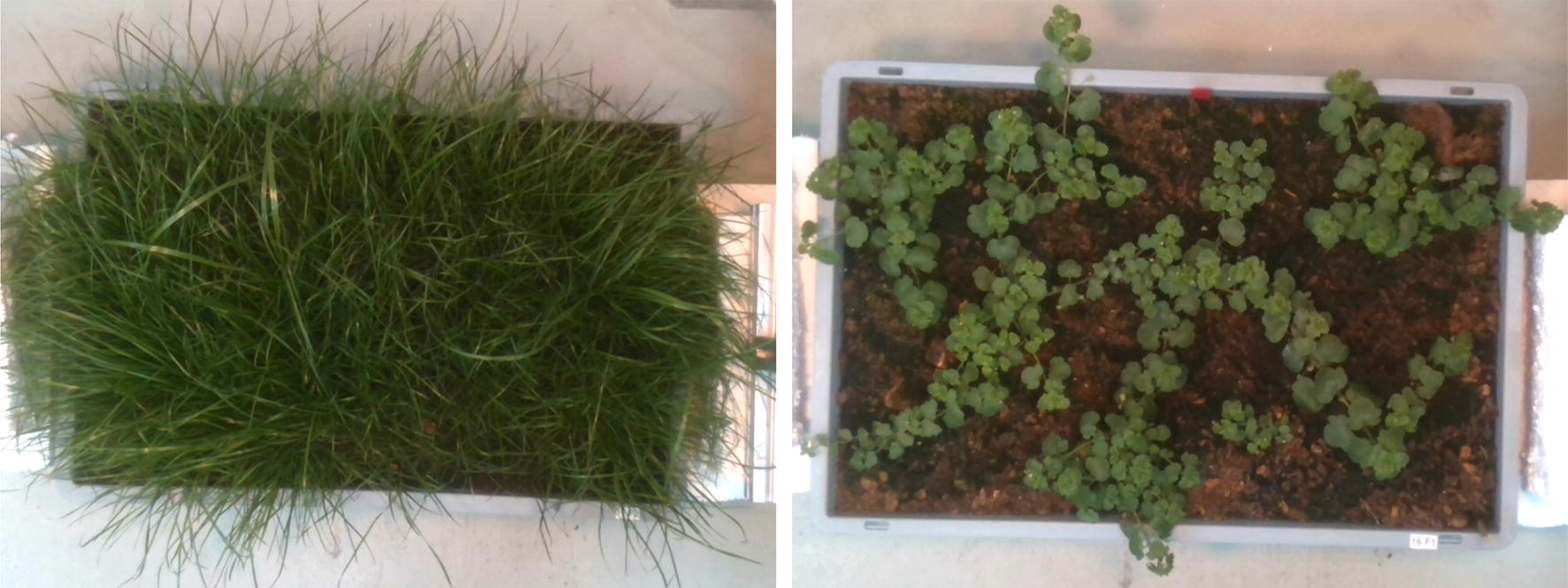
Images of monocotyle and dicotyl weeds used to create weed stress during the experiment

To establish weed pressure we used monocotyle and dicotyl weeds, shown in Fig. 2. As monocotyle weeds we used three grass species—*Poa pratensis* L., *Lolium perenne* L. and *Festuca rubra agg.* L., in variable combinations as derived from the mulch meadow grass seed mixture provided by Ufa Seeds (fenaco, Bern, Switzerland) [35]. As dicot species we used locally collected *Stellaria media* (L.) Vill. (common chickweed). We established three levels of weed density: no weeds, medium weed pressure containing 2–4 chickweeds (without grass) and a high weed pressure containing 4–8 chickweed plants and 2–4 grasses totalling in 7–12 weed plants per cultivation box. The weed pressure classes medium and high were established according to experience from previous field and greenhouse experiments. Additionally, we established boxes of single and mixed weed species without sugar beet which may be useful for classifier training purposes.

#### Water (drought) stress

**Fig. 3:**
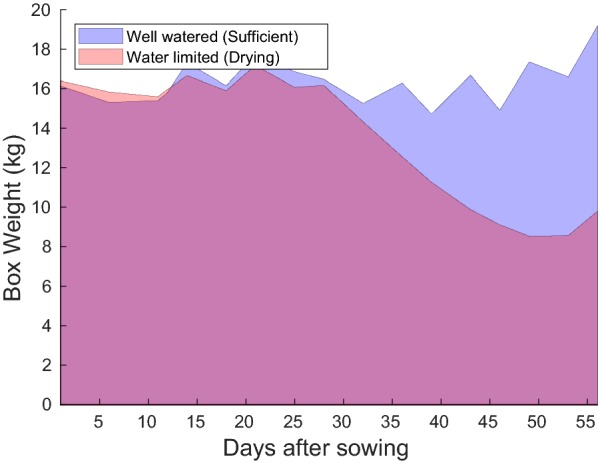
Area plots of box weights over time for water limited and well watered boxes used for creating the two levels of water stress during the experiment. The reduction in box weight in the water limited treatment from 28 days after sowing (DAS) onwards reflects the reduced soil moisture causing drought stress

We established two severity levels of drought stress. To the water limited (drying) plant boxes, we provided an initially sufficient water supply which was followed by a drought phase after germination. The well watered plant boxes were kept well irrigated by regular subsequent re-watering every 2–3 days. A detailed timeline of all treatments is available with the dataset [1]. For the limited water supply treatment irrigation was withheld starting from 14.02.2018 and regular watering started again at 12.03.2018. The boxes were weighed at the beginning and on every measurement date during the experiment to provide a reference measurement directly corresponding to soil moisture content. A sample plot of box weights for the sufficient and limited water supply treatments can be seen in Fig. 3.

An overview of the different stress treatments applied to the plant cultivation boxes under study during the experiment is provided in Table 1.

### Imaging setup

**Fig. 4:**
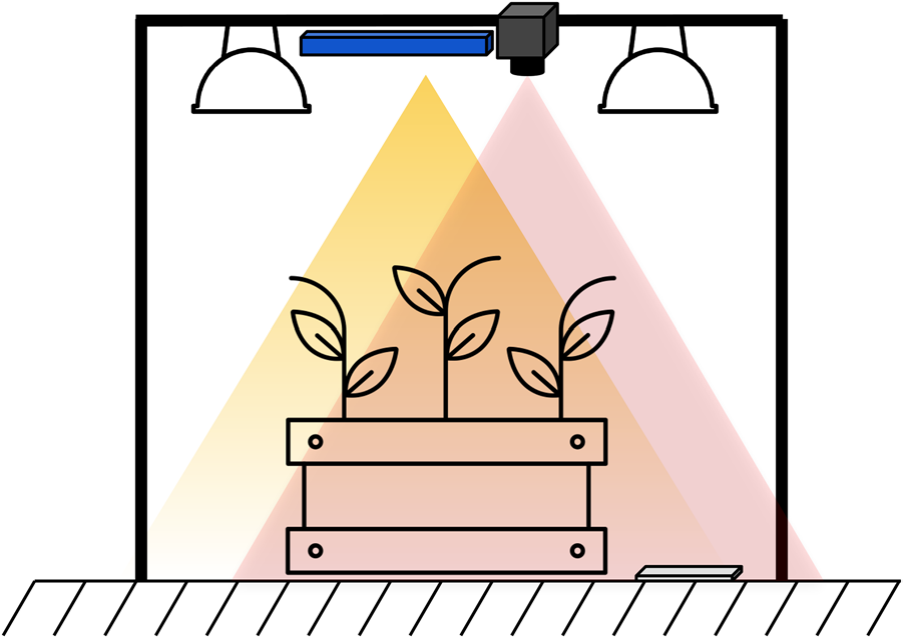
Schematic of the experimental setup. The Intel ZR300 (blue) and the Ximea Hyperspectral camera (gray) were the primary imaging sensors used for the remote measurements. The two sensors were mounted on a frame constructed using item® profiles. Two halogen lamps were mounted on either side of the sensors to ensure sufficient illumination in the visible and near infrared range of the cameras. A reference reflectance panel was placed in the field of view of both cameras for each image

**Fig. 5:**
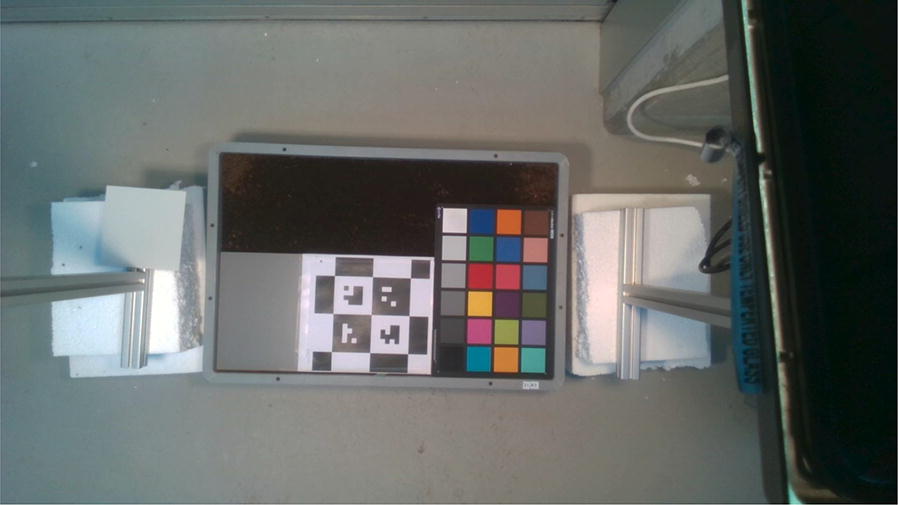
A reference cultivation box with a x-rite ColourChecker® chart and a CALTag marker, for geometric and radiometric calibration was imaged on each measurement date. This enriches the dataset by allowing for the possibility of recalibration of both the geometric and radiometric parameters for the two sensors

The measurement setup used to collect the dataset consisting of biweekly measurements after germination of the 30 boxes planted with sugarbeet and/or weeds and the treatments described above is depicted in Fig. 4. Imaging is done using two sensors, the Intel®Realsense ZR300 camera and the Ximea MQ022HG-IM-SM5X5-NIR Snapshot Hyperspectral camera. These sensors were chosen since they satisfy the criteria of being both light weight and low power while being able to provide accurate multi-modal data under outdoor conditions. This makes them ideally suited for deployment on the field onboard hand-held sensors, smart tractors, UAVs and UGVs alike. Both the sensors were mounted on a frame constructed with item profiles and their locations and field of views optimized to overlap while imaging the boxes as depicted in the schematic. A reflectance panel with a homogeneous reflectance of 0.6 over the 400–1000 nm wavelengths was placed within the field of view of both sensors for radiometric correction of the hyperspectral data. In addition to the plant boxes, an additional box, depicted in Fig. 5, containing a x-rite ColorChecker® chart and a Caltag [36] marker was imaged on every measurement date in order to allow for radiometric and geometric recalibration of the cameras if and when required. The plant boxes were placed such that the soil surface was approximately 1 m below the cameras resulting in a raw image ground sampling distance of 0.72 *mm/pixel* for the color camera and 1.7 mm/pixel for the hyperspectral camera.^1^ Custom software drivers were developed using the manufacturer provided software development kits (SDKs) for the cameras in the setup to enable simultaneous triggering of all cameras during image acquisition.

### Sensors

#### Intel Realsense ZR300

The Intel® RealSense™ ZR300 camera [37] consists of a 2 MP rolling shutter color camera, an infrared camera pair for depth imaging up to 3.5m, a 6 degree of freedom inertial measurement unit, and a fisheye optical sensor in a single module with a USB 3.0 interface for both power and data transfer. We used the *k*alibr.^2^ [38] framework for estimating the cameras’ intrinsic parameters-focal length, principal point and radial-tangential distortion coefficients. The *k*alibr framework was also used for extrinsically calibrating the cameras w.r.t each other.

#### Ximea snapshot hyperspectral camera

**Fig. 6:**
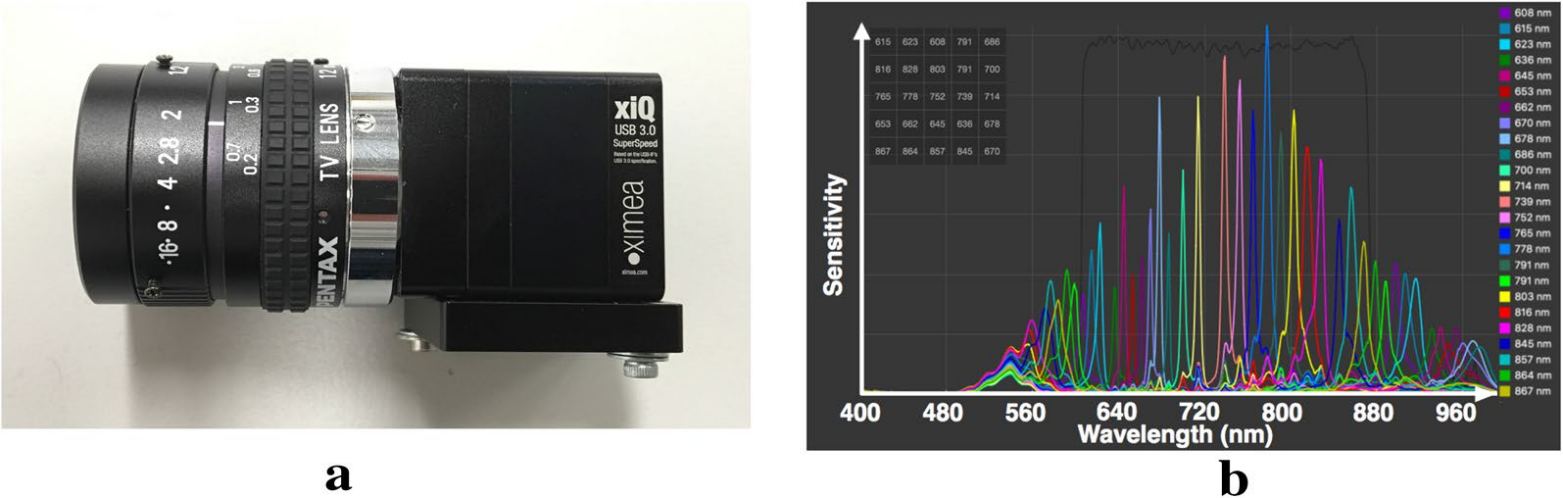
Ximea snapshot hyperspectral Camera MQ022HG-IM-SM5X5-NIR with Pentax C61215TH 12 mm lens (**a**) and manufacturer specified spectral response of the hyperspectral sensor (**b**)

The Ximea MQ022HG-IM-SM5X5-Near Infra-red (NIR) Snapshot Hyperspectral (HS) Camera [39] (Fig. 6a) consists of a 2 MP global shutter CMOS imaging sensor with Fabry-Perot interferometric spectral filters placed directly on top of every image pixel. The camera spectral range consists of 25 narrow-band channels from 600 to 950 nm. This implies a 409 × 216 pixel resolution image for each of the 25 channels of the camera (without interpolation). The camera also provides a USB 3.0 interface for power and data transfer. Images may be acquired at up to 170 frames/second. An IR short pass filter which blocks wavelengths above 875 nm was mounted on top of the camera lens resulting in the sensor response curve shown in Fig. 6b for the 25 camera channels. The exposure time of the camera was adjusted to avoid saturation and fixed at a value of 50 ms throughout the measurements. The camera and lens system was geometrically calibrated using the *k*alibr framework and radiometrically calibrated using the method described in [40].

### Dataset description and reference traits

**Fig. 7:**
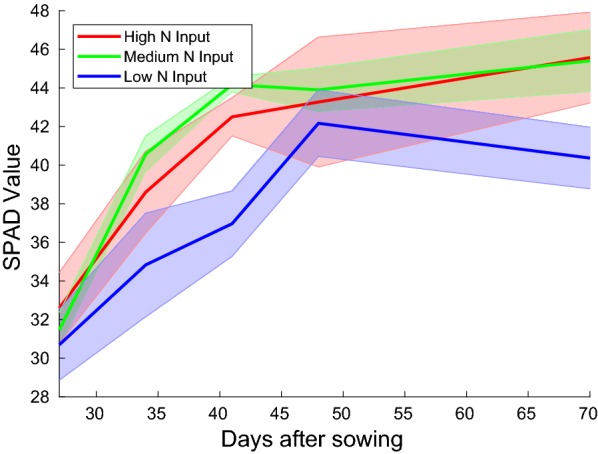
Means and standard deviations of SPAD measurement values obtained over three replications of each of the three different Nitrogen treatment regimens. One can see that the plant N availability which correlates strongly with the SPAD values is lower in the deficient (Low N input) case, however, similar for the medium and high N input levels. This indicates that the plants’ Nitrogen requirement in these two cases is satisfied, hence there is little need for the additional fertilizer present in the high N treatment

A total of 1984 images were captured (31 boxes × 16 dates × (1 RGB $+$ 2 IR $+$ 1 HS)) along with temperature and humidity data from the greenhouse control system every 12 min for the duration of the experiment. As a reference measurement to track plant N status we used a chlorophyll meter SPAD-502PLUS [41] reflecting the chlorophyll content of a leaf which is strongly related to the N supply of a plant. Weekly SPAD measurements for each cultivation box were conducted after the leaves grew to a measurable size, by averaging measurements from the youngest fully developed leaf of each of the six plants per box which. The means and standard deviations of the SPAD values measured over three replications of each of the three different Nitrogen treatments is plotted in Fig. 7. To track soil moisture content, box weights (water, plus substrate, plus plant biomass) were measured along with the imaging measurements. Following the harvest on the 29.03.2018, above and below ground plant biomass was weighed for fresh weight (FW) directly after harvest and for dry weight (DW) after drying at $60^{\circ }\hbox {C}$ until it achieved a constant weight in a drying oven.

### Data pre-processing and plant trait extraction

This section describes the data pre-processing algorithms and methods for extracting plant trait indicators from the collected dataset. Documented MATLAB® code with the pre- and post-processing methodology described in this section is available online [2].

**Fig. 8:**
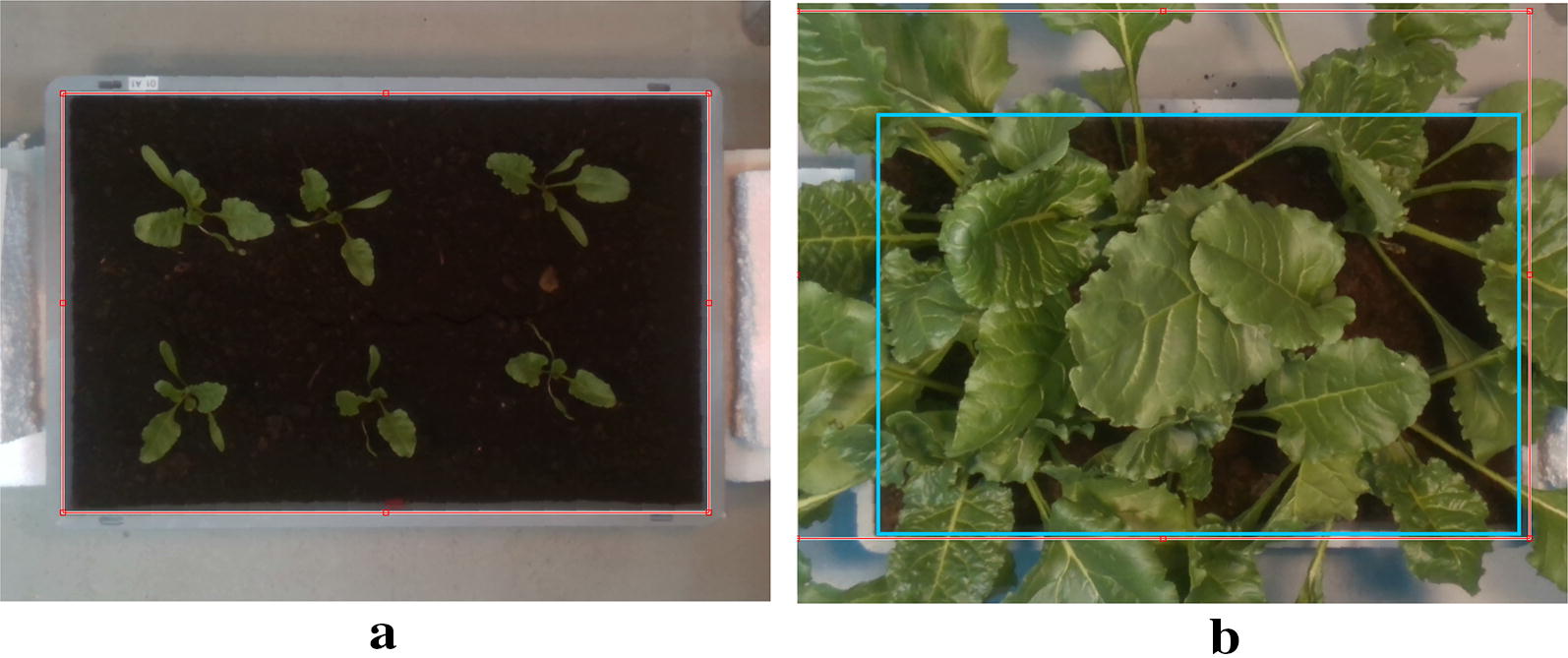
User interface for semi-automated box (ROI) detection. The user is provided an interface to verify the automated detection (**a**) and is able to modify it in cases where the automated detection is not satisfactory (**b**). The cyan rectangle in **b** depicts the user corrected ROI actually used for further analysis

#### Region of interest(ROI)/box detection

In order to facilitate extraction of relevant parts of the images and include only the foreground, i.e the boxes containing the plant and soil material, we provide a semi automated work flow for detecting box boundaries in the RGB images. We rely on observing similarly colored gray points on the edges of the box and then use one of the two procedures described below:

1. Fit the box boundaries to the most prominent detected edges, i.e locations of highest edge point density. This method is relatively robust to outliers, however works well only if lots of points on the box edges are observable in the image, which is only the case for early growth stages with low canopy cover.
2. Span a rectangle of maximum size, that does not contain any of the edge points. This method only needs few (e.g. 2 per boundary) points in order to detect a rectangle, and thus works well even in cases of extreme coverage by the canopy. However, it is sensitive to outliers detected within the box (e.g plant or soil pixels detected as edges since they appear “gray” in the image).

The automated detection candidates are then provided to the user for feedback through a pop-up window (Fig. 8), where the user can drag the box outlines to the correct box edges (Fig. 8b). Once the box areas are detected in each of the color images, they are saved for further processing steps. We release our annotations based on this work flow for the pixel positions of the box corners for each image along with the dataset.

#### Reflectance computation

Radiometric calibration for the hyperspectral camera is performed using the method described in [40]. Furthermore, a plate of uniform reflectance across the wavelength range of interest is placed in the measurement setup to provide a normalization factor under varying illumination conditions. The reflectance normalization factors, $f_{reflectance}$, are estimated using the reflectance plate in every image with known constant reflectance of *R* = 0.6. Since the plate is fixed with respect to the cameras, its location in the images is always the same. The reflectance factors are defined as the ratio of point reflectance to its observed intensity

1 $$\begin{aligned} f_{reflectance} = \frac{R_{pixel}}{I_{pixel}} \end{aligned}$$

Since the camera exposure time and white balance is set to utilize the full 10 bit range of digital number values provided by the camera, the pixel intensity values do not directly correspond to the point reflectance. Assuming uniform illumination over the area of interest, a robust reflectance factor can be estimated from a region of known reflectance, i.e. the reflectance panel, using

2 $$\begin{aligned} f_{reflectance} \approx \underset{pixel \in Region}{{\text {mean}}} \left( \frac{R_{pixel}}{I_{pixel}}\right) \end{aligned}$$

independently for each channel (wavelength band). Occlusions of the reflectance panel, however, remain a potential source of error. To overcome errors due to occlusion, at first, occlusions of the reflectance panel are detected in the RGB image, since it is easier to find values deviating from the expected brightness. If the reflectance panel region contains any pixels, darker than a certain threshold (0.4 in this case), the image is marked as occluded. The reflectance factors are then computed for all non-occluded images in the dataset and all image types (RGB, IR and HS). The reflectance factors for occluded images are approximated by averaging the first non-occluded images taken before and after the image of interest. If there are no non-occluded images before, only the following image is considered. In the worst case, this procedure leads to assuming the reflectance factor from the reference box image (Fig. 5), where the reflectance panel is always fully visible.

#### Spectral 3D point cloud generation

**Fig. 9:**
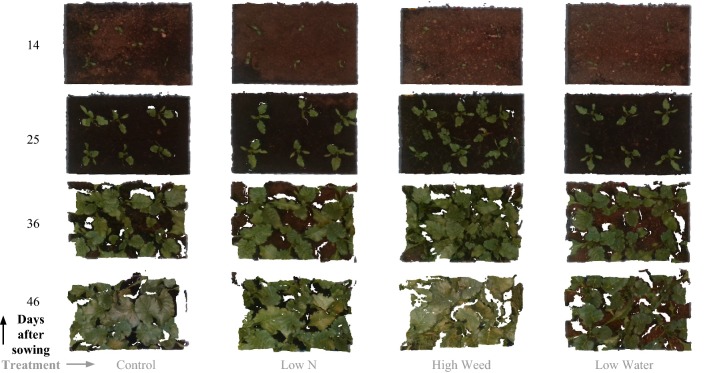
Processed colored point clouds of four selected treatments at four consecutive measurement dates (same as Fig. 10) extracted using our pipeline. These top down rendered views depict the structural change between the plants exposed to different stress treatments over time. It can be seen from these rendered views that discerning differences between the different treatments is difficult using only RGB style images, hence a multi-modal approach is desirable, providing a rich variety of data sources to create indicators encompassing visual, geometric and spectral information

**Fig. 10:**
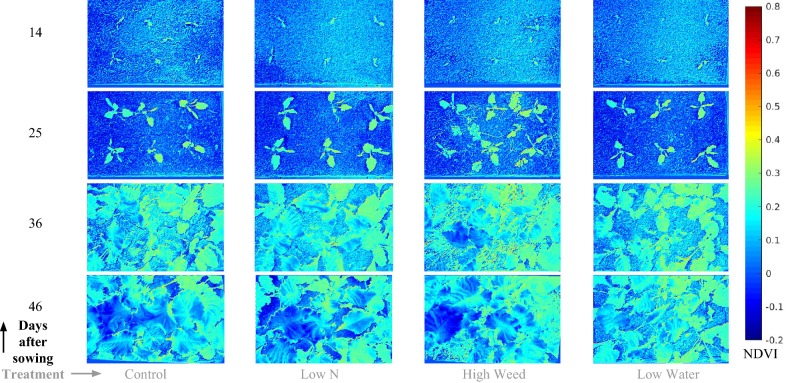
Calibrated NDVI images for the same dates and treatments as Fig. 9. The near infrared (*NIR*) and red (*R*) wavelengths used correspond to the 803 nm and 670 nm bands. The availability of 25 narrow-band reflectances within the dataset, enables the possibility to systematically study plant vitality and growth over time under the different stress factor-severity level combinations and their effect on many multi-channel vegetation indices

We combine the information from the color, stereo-infrared and hyperspectral images to create spectral point clouds for each image set. This data structure is fundamental to all further processing steps including plant trait indicator computation and classification. The spectral 3D point cloud stores information about each box on each measurement date in the form of a point cloud, where each point is defined by its 3D cartesian coordinates w.r.t the left IR camera optical center, RGB colors and spectral reflectance data. The point clouds are constructed as follows:

1. The two infra-red images are used to extract the 3D structure of the scene in the form of a point cloud using the stereo processing approach described in [42].
2. Each 3D point in the above point cloud is then associated to a pixel in the color image by projecting its 3D coordinates into the color camera using the extrinsic camera calibration parameters [43]. The RGB values of the corresponding pixel can then be associated with the 3D point, producing colored point clouds, such as the ones shown in Fig. 9.
3. A similar procedure is followed to project the 3D point into the hyperspectral image, thereby associating the corresponding 25 narrow band reflectance values with this 3D point. These reflectance values can then be used to produce phenotyping related index maps (e.g NDVI, NDRE), such as those shown in Fig. 10, which can be used to follow the development of physiology and chemistry (e.g chlorophyll levels [44]) within the plants over time.

#### Vegetation segmentation

Vegetation segmentation is an important pre-processing step which effects all derived plant trait indicators. In contrast to previous popular approaches where segmentation is performed using only one modality such as thresholding in some colour space [45, 46] or based on some index [47], we perform robust segmentation using a combination of color, hyperspectral and spatial cues. Empirically determined thresholding criteria for each of the three sensing modalities were found to provide excellent segmentation quality over the entire range of illumination conditions, growth stages and plant species present in the dataset. All points matching one of the following criteria were classified as vegetation:

3 $$\begin{aligned}&2G-R-B \ge 0.08 \end{aligned}$$

4 $$\begin{aligned}&R_{857nm} - R_{686nm} \ge 0.35 \end{aligned}$$

5 $$\begin{aligned}&Height \ge 0.02 \end{aligned}$$

**Fig. 11:**
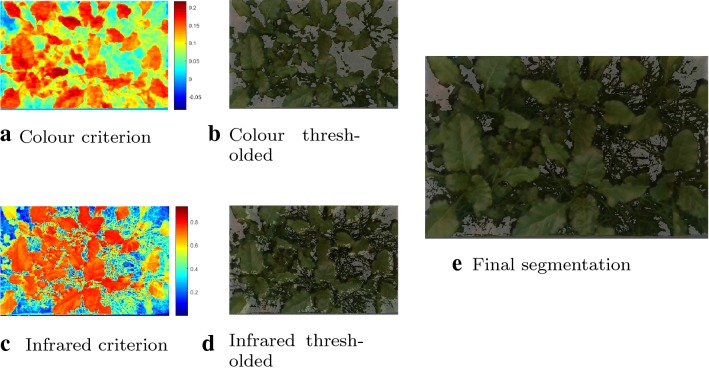
Visualization of the segmentation process. In **a** and **c** the criteria from Eqs. 3 and 4 are depicted using a jet colour map, **b** and **d** show the corresponding thresholded images. **e** contains the final combined segmentation projected onto a colour image

The Excess Green Index (Eq. 3) is incorporating information from the color images, where R, G and B are the reflectance of the color channels. Equation 4 includes information from the hyperspectral images by comparing infrared to red reflectance. Equation 5 adds height information to include points which may be incorrectly classified by the above two criteria due to shadows. The three modalities are fused in order to include sufficient information from all sources into the segmentation. This process is depicted in Fig. 11.

### Remote trait indicator based stress severity classification

#### Classifier training methodology

The provided dataset contains a total 16 measurement dates over the growing period for each of the 30 boxes with different treatments listed in Table 1. Since the first measurement date (day of sowing), does not contain any germinated plants, it was removed for this analysis. The remaining data was split into a training and test set:- all measurements pertaining to 6 randomly selected boxes (20% of the total data) were not available to any of the classifiers during training, in order to serve as an independent and unbiased test set.

In order to evaluate the utility of the dataset with respect to automated stress inference, we trained classification models using a variety of commonly used machine learning techniques using the spatio-temporal spectral feature set listed in Table 2 created from the indicator-statistic pairs described in section "Temporal evolution of plant trait indicators". Each feature (e.g. Height, Canopy cover, reflectance) was standardized by scaling it to zero mean and unit variance across the training set. With the time parameter appended, in units of days after sowing (DAS), to the 54 indicator-statistic pairs after scaling, the input to the models were 55 dimensional vectors, with components enumerated in Table 2. Given these input vectors, the classifier models were tasked with predicting the level of severity for each of the three stress factors. The different levels for each of the three stress factors, i.e the possible output classes of the classification model are listed in Table 3. Separate classification models were trained for each of the 3 stress factors.

We employed five-fold cross-validation for training the classification models. In *k*-fold cross-validation, the training dataset is randomly separated into *k* equally sized *folds* or groups. From the *k* groups, a single group is used as the validation set for testing the model predictions by comparing them to their true values, and the remaining $k-1$ groups are used as the training data. The process is then repeated *k* times, with each of the *k* groups used once as the validation set. The *k* results can then be averaged to obtain a mean cross validation accuracy. Cross validation allows one to assess the general applicability of a classification model, by preventing over-fitting on the training data. Furthermore, our classification scheme warrants non-uniform misclassification costs, since misclassifying a high nitrogen or weed stress sample as a medium one is more appropriate than misclassifying it as a low one. To take this factor into account we define misclassification cost matrices, as shown in Table 4 for the Nitrogen and Weed stress classifiers.

#### Classifier performance evaluation

**Fig. 12:**
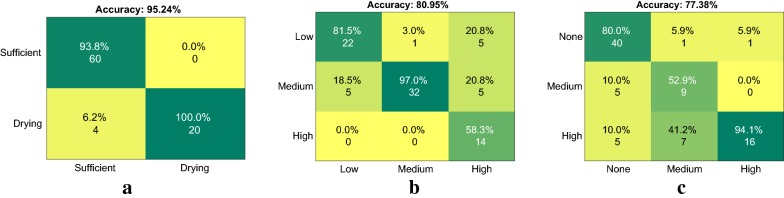
Confusion matrices for the SVM classifier models on the test dataset for **a** Water Input, **b** Nitrogen availability and **c** Weed pressure. The rows correspond to the true target class and the columns to the predicted class labels. The entries of the matrix show the percentage accuracy for each target-prediction combination followed by the number of query observations will fall into that category

We evaluated several machine learning methods, listed in Table 5 in order to train the classifiers which learn a function (mapping) from the input feature vectors (Table 2) to the output class (Table 3), i.e predict the level of severity for each of the three stress factors under study using the plant trait indicators extracted from the images. We used the confusion matrix [48] to evaluate and compare the performance of the different classification methods. The entry in the *i*th row and *j*th column of a classifier’s confusion matrix, $CM_{ij}$ contains the total number of observations for which the actual class is *i* and the predicted class is *j*. The accuracy for each of the methods quantifies the fraction of the training dataset which is correctly predicted by the trained model. The mean misclassification cost can be computed as

6 $$\begin{aligned} cost = \frac{1}{N}\sum \limits _{i}\sum \limits _{j} CM_{ij}w_{ij} \end{aligned}$$

where $CM_{ij}$ and $w_{ij}$ are the corresponding entries in the confusion and misclassification cost matrices respectively and *N* is the total number of observations or predictions. As seen from Table 5, several classifiers show high accuracy when trained with the entire set of multi-modal features. From this analysis, the SVM classifier with a quadratic kernel shows the highest cross validation and test set accuracy from the tested methods, indicating good generalization. The confusion matrices for the trained SVM models on the test dataset are shown in Fig. 12. This model is quick to train and multiple hundred predictions can run in real time on a robot or high throughput phenotyping system if required. Please note the main objective of this section is to present a baseline based on data driven approaches, and a thorough comparison of machine learning approaches is beyond the focus of our evaluations.

**Table 2:** Indicator-statistic pairs used as features for training the machine learning based classification models

Indicator	Statistic
Canopy cover	Mean
Volumetric estimate	Mean
Height	Mean and variance
MS* (all 25 channels)	Mean and variance
Days after sowing	–

**Table 3:** Stress factor-level pairs listing the possible output classes for this dataset

Stress factor	Severity level
Water input	Sufficient, limited
Nitrogen input	Low, medium, high
Weed pressure	None, medium, high

For an ideal classifier, given input features extracted from a plant box, the output classes should correspond to the provided treatments listed in Table 1. For the water stress classifier, the correct target label was “Sufficient” for all boxes till 28 days after sowing and “Drying” for the boxes with limited water supply afterwards (Fig. 3)

**Table 4:** Non uniform misclassification cost matrix for the nitrogen and weed stress classifiers

	Predicted class		
	Low/none	Medium	High
*Actual class*			
Low/none	0	1	2
Medium	1	0	1
High	2	1	0

**Table 5:** Mean cross validation accuracies for different machine learning algorithms on the dataset [2]

Method	Cross validated training accuracy			Test set accuracy		
	Water	Nitrogen	Weeds	Water	Nitrogen	Weeds
Decision trees [49]	93.39	63.66	60.36	86.90	47.62	65.48
$\hbox {LDA}^{\mathrm{a}}$ [50]	96.10	68.47	75.68	94.05	78.57	75.00
${{SVM}}^{\mathrm{b}}$ [51]	93.09	75.68	83.18	95.24	80.95	77.38
$\hbox {KNN}^{\mathrm{c}}$ [52]	92.79	62.16	69.97	92.86	55.95	65.48
BaggedTrees [53]	94.89	67.57	71.47	91.67	63.10	69.05
Subspace discriminant [54]	94.59	70.57	75.08	94.05	75.00	72.62
Subspace KNN [54]	93.39	60.66	64.26	97.62	66.67	72.62
$\hbox {RUSBoostedTrees}^{\mathrm{d}}$ [55]	95.20	69.37	69.37	97.62	63.10	71.43

For detailed descriptions of the machine learning methods evaluated we refer the reader to the cited papers. The SVM classifier showed the best overall performance from the tested methods, on both the training and test data, indicating good generalization to novel inputs. The implementations for the classification methods provided by the MATLAB® Statistics and Machine Learning Toolbox were used. The specific parameters for each of the classifiers can be found within the MATLAB® functions provided in the accompanying software suite

${}^{\mathrm{a}}$ Linear discriminant analysis

${}^{\mathrm{b}}$ Support vector machine

$${}^{\mathrm{c}}$$ $$k-$$nearest neighbor

${}^{\mathrm{d}}$ Randomly undersampled boosted trees

## Results and analysis

### Effect of stress on yield

**Fig. 13:**
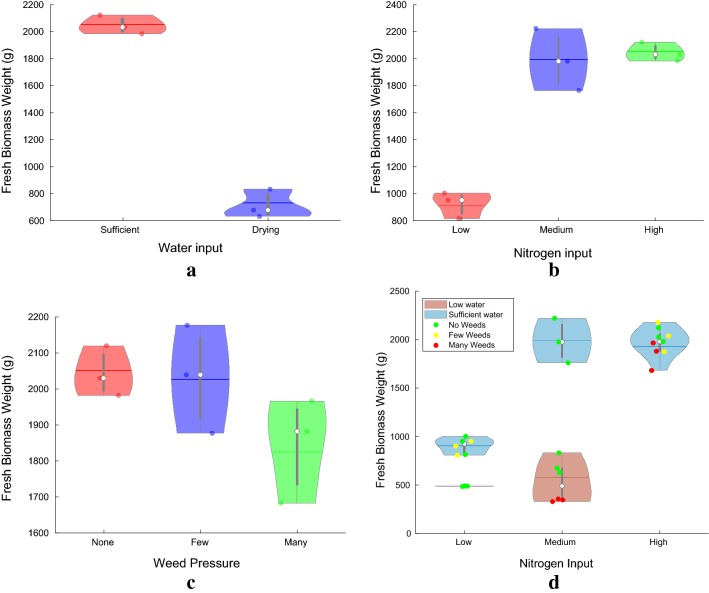
Violin plots visualizing the effect of the different stress factors: **a** water limitation, **b** N availability, **c** weed pressure and **d** their interaction, on fresh biomass weight measured at the conclusion of the experiment. The bold line in the middle of each lobe represents the mean, the white dot is the median and the lobe extents along the Y-axes are the 95% confidence intervals for the data corresponding to each lobe. The total fresh biomass weight including both above and below ground biomass serves as a good proxy for plant performance, stress reaction and subsequently for yield potential. The interaction plot **d** shows the relative impact of the three different stress types. Under the conditions established during this study, water limitation has the highest impact on yield, followed by Nitrogen availability and then weed pressure

In order to study the impact of the different treatments on beet yield, the beet roots were extracted and weighed at the completion of the experiment. Additionally, the dataset also contains dry and fresh weights of the above ground biomass (leaf material). The three investigated stresses, N deficiency, weed pressure and water limitation had varying impact on the total biomass production (Fig. 13). The fresh biomass weight at the conclusion of the experiment i.e the sum of both above (shoot) and below (root) ground biomass is used here as a proxy for the yield potential since the plants did not reach maturity. Figure 13a–c depict the variation in yield as a function of the three stress factors-water, nitrogen and weeds, respectively, while the levels of the other stress factors was kept constant for each case.

One can see the adverse impact of drought stress on yield in Fig. 13a where the average fresh biomass weight per box drops from about 2000*g* to 700*g*, with sufficient Nitrogen availability and no weeds present. This is in accordance with the expectation that water limitation reduces biomass accumulation in sugar beet [56, 57]. From Fig. 13b we can observe that the medium level N treatment was found to be sufficient for the sugar beet growth during the two months of the experiment reflected by the increase in biomass production from approximately 900*g* to above 2000*g*, for the low and medium N fertilization treatment, respectively. On the other hand, additional N input supplied in the High N availability treatment had little yield increasing impact. This represents an optimal N supply level, which in field sugar beet production is very much related to extractable sugar yield, and is compromised by both too low and to high N fertilization levels [6, 32, 58]. Low weed pressure might be tolerated by a sugar beet crop but high weed pressure as created in this experiment reduces the sugar beet biomass production (Fig. 13c). Under field conditions such competition with weeds has also been reported to have lead to reduced sugar yield [59, 60]. One insight that can be derived from these stress reaction plots (Fig. 13a–c) is that *under the stress conditions established during this experiment, drought stress had the most severe impact on fresh biomass yield, followed by Nitrogen availability and then weed pressure*.

Figure 13d clearly shows that that under realistic field conditions, stresses occurring simultaneously interact with each other and have a combined effect on biomass production and yield. These interactions make the determination of the individual stresses and their severities using remotely sensed data more complex. However, since different stresses affect yield by different amounts, having data about how each stress factor affects yield independently along with the impact of certain combinations should make this task computationally tractable.

### Temporal evolution of plant trait indicators

**Fig. 14:**
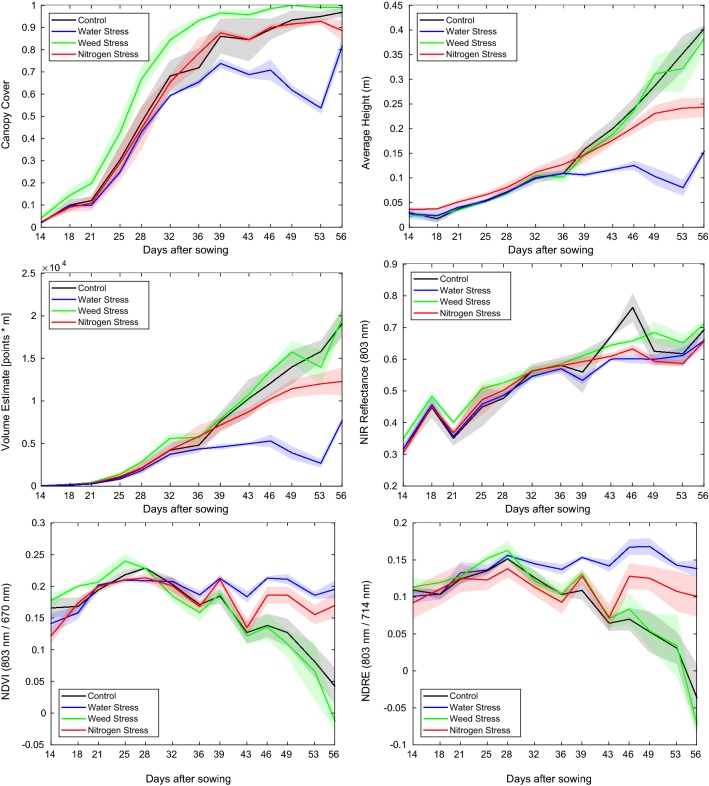
Development of selected plant trait indicators over time for the control group as well as those subjected to the three abiotic stress factors. The translucent regions indicate one standard deviation from the mean for cultivation boxes subject to identical treatment conditions. The temporal evolution of two popular vegetation indices—Normalised Difference Vegetation Index (NDVI) and Normalised Difference Red-Edge Index (NDRE), averaged over the biomass in the boxes is also shown. The control group as well as plants subject to weed stress are observed to follow the standard phenological development [63], whereas the resource constrained plants do not reach senescence during the experimental period

We refer to plant trait indicators as numeric values, representing a spatial or spectral property of plants associated with an image patch, in this case of the box. They are extracted from the calibrated spectral point clouds by taking a statistical measure, such as the mean or variance of the quantity of interest over all points labeled as vegetation. Examples of useful plant trait indicators would be the total canopy cover, average height and normalized narrow-band reflectances. Based on the vegetation segmentation, described in section "Data pre-processing and plant trait extraction", a set of plant trait indicator values, listed in Table 6 is extracted from the spectral point clouds. These plant trait indicators can be used to monitor the development of the plants and observe the effect of the different stresses on multiple aspects of plant growth (Fig. 14). A variety of effects may be observed from the plots, such as:

- the canopy cover for weed infested boxes is significantly higher than the boxes with no weeds and the vegetation canopy closes earlier
- drought stress has a significant affect on plant height. The affect of stopping regular water supply at 28 DAS becomes apparent in the average plant curve at 36 DAS.
- the NIR reflectance of the boxes under Nitrogen stress is slightly reduced compared to the control group.

Since we have shown that the three stress factors affect yield (section "Effect of stress on yield"), one of the goals of remote stress phenotyping is to find remotely detectable plant trait indicators which allow the differentiation of these stress factors. The presented plots indicate that indeed such indicators or indicator combinations may be found using a combination of spatial, spectral and temporal cues. The identification of such plant trait indicators can be supported by using a variety of statistics from the point clouds, such as those listed in Table 7. An Analysis of Variance (ANOVA) procedure may be used to detect statistically significant differences, corresponding to the stress treatments, within these remote trait indicators. Such analyses are made simple and straightforward through the provided dataset and accompanying software and may be used to find promising indicators and their combinations which are indicative of particular stress factors, which are not only limited to this dataset but generalize to the field as well. Plant growth is typically faster under greenhouse conditions and, higher plant density in the boxes than in the field results in comparatively faster canopy closure. However, in field trials, it is typically difficult and expensive to accurately control soil conditions such as N availability and water content. The greenhouse dataset, with defined nutrient and water inputs, provides an ideal test bed to establish and validate frameworks for determining probable causes of stress from remotely measured data using statistical machine learning tools. Such a framework can then be deployed on the field after data augmentation from conducting much smaller and hence inexpensive field trials.

**Table 6:** Selected plant trait indicators which may be computed using the collected dataset and provided post processing software

Acronym	Full name (unit)	Description
Height	Height (m)	Height (=Z coordinate) above the soil reference height. Negative values are ignored
NDVI	Normalized difference vegetation index (−)	Defined as $(NIR-R)/(NIR + R)$, where NIR is the near infrared (803 nm)and R is the red (670 nm) reflectance respectively [61]
HS*	Hyperspectral reflectance (−)	Hyperspectral reflectance, where *$\in \{1,\ldots , 25\}$ indicates the spectral band index
EGI	Excess green index (−)	Defined as ($2G-R-B$), based on the color reflectance [62]
NEGI	Normalized excess green index (−)	EGI divided by $(R + G + B)/3$
NDRI	Normalized difference red index (−)	Defined as $(G - R)/(G + R)$, based on color values
ERI	Excess red index (−)	Defined as $1.4R - G$, based on color reflectance
HSDiff*$^1$_*$^2$	Difference in hyper spectral reflectance (−)	Difference in reflectance of two hyperspectral bands *$^1$–*$^2$, where *$^1$, *$^2$ $\in \{1,\ldots , 25\}$ indicates the spectral bands
CanCov	Canopy cover (−)	The percentage of points labeled as plants. Canopy cover is independent of the evaluation statistic
VolEst	Volumetric estimate (m*pixels)	The integral over the height of all points. Volumetric estimate is independent of the evaluation statistic

Due to the objective oriented nature of the software suite and comprehensive spatio-temporal nature of the data provided, users of the dataset can readily implement additional indicators of interest such as multichannel vegetation indices, temporal indicators e.g rate of growth for further analysis

**Table 7:** Supported statistics which may be computed for each box for the trait indicators listed in Table 6

Statistic	Description
Mean/m	Average over all points
Variance/v	Variance within all points
Min	Minimum attained value
Max	Maximum attained value

### Impact of input feature vector modality on classifier performance

**Fig. 15:**
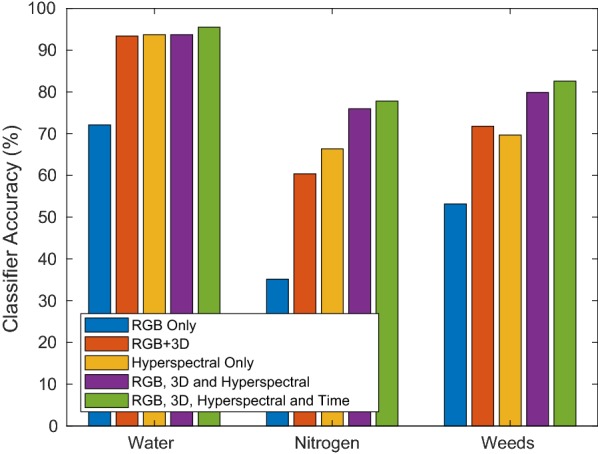
Comparison of classifier cross validation accuracy as a function of the input feature set used. One can observe that the multi-modal nature of the dataset provides the highest overall classifier performance. One can also observe how different kinds on features influence the prediction performance for different stress factors, e.g. the 3D data is an important indicator of weed pressure due to the significant height difference between the plants and weeds, resulting in a lower mean height while increasing the variance compared to the control group

Figure 15 shows the impact of input feature modality on classification performance for a SVM classifier. The RGB only classifier was trained using features that can be extracted using only the color imagery (i.e Canopy Cover), the RGB+3D classifier using features from color and stereo infrared imagery (i.e Canopy Cover, Height, Volume), the Hyperspectral only classifier was trained on data from the hyperspectral camera (i.e the 25 HS reflectances), the RGB+3D+Hyperspectral classifier using features from all of the above modalities and the RGB+3D+Hyperspectral+Time classifier also had access to the time (in DAS) for each measurement. It can be observed that the spatio-temporal spectral combination of color imagery, 3D data, hyperspectral reflectances and time outperform the individual modalities, for each of the stress factors.

## Conclusions and outlook

In this work we have provided a multi modal framework for systematically studying the effect of drought, nitrogen deficiency and weed stress on plant growth. Our framework includes a dataset containing remotely sensed data measured in a greenhouse, which may also be measured with an unmanned aerial or ground vehicle on the field as well as associated standard reference measurements which are typically manually measured for evaluation and benchmarking. Furthermore, we included a pre- and post-processing software framework along with the dataset which includes functionality for radiometric normalization, 3D point cloud extraction, plant trait extraction and machine learning based stress severity level classification. We showed an effective, generic and plant agnostic methodology for feature extraction and machine learning based stress severity level classification from multi-modal remotely sensed data, which can be readily applied to a wide variety of crops. We also showed that remotely measured spatio-temporal spectral plant trait indicators can indeed be used to accurately and simultaneously predict the presence and severity of multiple stress factors which is the predominantly occurring condition on the field. This will pave the way for automated, timely, effective and precise intervention actions in order to maximize yield while minimizing environmental impact and additional resource input.

There are many interesting avenues for future work. For example, predicting yield based on the spatio-temporal remote sensing data, using ground truth biomass measurements which are available within the provided dataset. The precision and accuracy, especially for Nitrogen and Weed stress severity level classification, may be further improved by collecting larger datasets. Additional sensing modalities, such as thermal reflectance and fluorescence can be added to the combination of the feature vectors input to the machine learning models when available. These additional modalities can also be readily analyzed using the functionality provided by the accompanying software. Since the software suite supports indicator ranking, the framework may also be extended to allow for a systematic evaluation of the most predictive features related to a particular stress factor. This would allow, for example, the selection of the most appropriate wavelength bands and spectral vegetation indices for Nitrogen availability measurement, supporting the development of dedicated lower cost sensors for detection of particular stress factors in both controlled and field situations.

## Footnotes

1 Note that since the hyperspectral camera has a 5 × 5 filter pattern, the GSD per band without interpolation is 5 times higher, i.e 8.5 mm/pixel.

2 Available at https://github.com/ethz-asl/kalibr.
